# The relation of unrest-related distress with probable depression during and after widespread civil unrest

**DOI:** 10.1017/gmh.2022.27

**Published:** 2022-07-20

**Authors:** Tiffany Junchen Tao, Tsz Wai Li, Sammi Sum Wai Yim, Wai Kai Hou

**Affiliations:** 1 Centre for Psychosocial Health, The Education University of Hong Kong, Hong Kong SAR, China; 2 Department of Rehabilitation Sciences, The Hong Kong Polytechnic University, Hong Kong SAR, China; 3 Department of Psychology, The Education University of Hong Kong, Hong Kong SAR, China

**Keywords:** Depression, objective intensity, unrest-related distress, social movements

## Abstract

**Background:**

This study investigated whether subjective unrest-related distress was associated with probable depression during and after the 2019 anti-ELAB movement in Hong Kong.

**Methods:**

Population-representative data were collected from 7157 Hong Kong Chinese in four cross-sectional surveys (July 2019–July 2020). Logistic regression examined the association between subjective unrest-related distress and probable depression (PHQ-9 ⩾ 10), stratified by the number of conflicts/protests across the four timepoints.

**Results:**

Unrest-related distress was positively associated with probable depression across different numbers of conflicts/protests.

**Conclusion:**

Unrest-related distress is a core indicator of probable depression. Public health interventions should target at resolving the distress during seemingly peaceful period after unrest.

## Introduction

In 2019, a controversial bill was introduced by the Hong Kong SAR Government for extraditing individuals accused of crimes to countries/regions which do not have a legal mechanism for doing so. The resultant anti-extradition law amendment bill (anti-ELAB) movement gradually escalated from peaceful demonstrations into massive violence and vandalism since June 2019. Protests diminished in early 2020 due to the coronavirus disease 2019 (COVID-19) but mounted again in May–June 2020 due to the hasty introduction of the National Security Law. The movement involved approximately 2000000 participants lasting over months, leaving serious mental health consequences (Holbig, 2020; Turnbull *et al*., 2020). The prevalence of depression rose from 1.9% a decade ago to as high as 25% in the heat of the movement (Ni *et al*., 2020; Hou *et al*., 2021). Given that depression forms one of the heaviest public health burdens and elevates the suicidal risks (WHO, 2021), it is pressing to further elucidate its development and maintenance under potentially traumatic events such as social unrests.

Risk factors of psychopathology embed within the event level (e.g. exposure, proximity) or the individual level (e.g. a lack of personal coping resources) (Galovski *et al*., 2016; Wong *et al*., 2021a). The anti-ELAB movement incubated, developed, and faded over a dynamic process, and this synchronized with the trend of depression over time (Hou *et al*., 2022). Subjective experiences of support and relationship quality with close social partners were positively associated with better mental health among adolescents during the anti-ELAB movement (Wong *et al*., 2021b). An experience sampling study illustrated that individuals experienced significant negative changes in their everyday emotions during a social movement, which predicted subsequent higher psychiatric symptoms and lower subjective well-being (Hou and Bonanno, 2018).

It is intuitive that individuals experience higher levels of stress with greater intensity of conflicts/protests, with direct or indirect exposure to street protests and related social media posts positively associated with elevated psychological distress, probable depression, and even suicidal ideation (Galovski *et al*., 2016; Turnbull *et al*., 2020; Hou *et al*., 2021; Lam *et al*., 2021). However, currently limited research has investigated whether the association between subjective unrest-related distress and psychiatric symptoms changes as a function of the intensity of unrest over time. There is also a deficit of knowledge about the comparative centrality of subjective experience of traumatization and objective facets of event exposures in understanding mental health consequences (Maschi *et al*., 2011; Boals, 2018; Su, 2018; Danese and Widom, 2020). Existing evidence is mainly based on recalls of objective and/or subjective traumatic experiences over a fraction of one's life history (Maschi *et al*., 2011; Boals, 2018; Danese and Widom, 2020), but less so following instant measures of civil unrest as an acute traumatic exposure.

This study aims to examine the association of subjective unrest-related distress with probable depression in Hong Kong between July 2019 and July 2020, a period with varying intensity of widespread conflicts/protests (Holbig, 2020). We also tested whether the positive association between unrest-related distress and depression remained the same across different number of conflicts/protests at different timepoints. This study hypothesized that unrest-related distress would be positively associated with probable depression. The positive association between unrest-related distress and depression would be consistent across different numbers of conflicts/protests over time.

## Methods

### Sample

This study was approved by the Human Research Ethics Committee of The Education University of Hong Kong. This study analyzed data from four population-representative samples of Hong Kong Chinese in serial cross-sectional surveys in July 2019 (T1), February 2020 (T2), April 2020 (T3), and July 2020 (T4) (*n* = 7157). The respondents were approached through random digit dialing by a Computer-Assisted Telephone Interview system, with contact numbers drawn from the Hong Kong Communication Authority databases. Inclusion criteria were: (1) Hong Kong Chinese, (2) aged ⩾15 years, (3) Cantonese speaking. Oral informed consent was obtained before each interview. The cooperation rates (i.e. completed over eligible) across the four surveys were 72.0%, 67.3%, 73.5% and 72.8%. More details were documented in prior studies (Lai *et al*., 2021; Hou *et al*., 2022). Demographics are summarized in Table 1.

**Table 1.** Descriptive information of the current sample at the four timepoints (*N* = 7157)

	T1 July 2019 (*n* = 1112)		T2 February 2020 (*n* = 2003)		T3 April 2020 (*n* = 2008)		T4 July 2020 (*n* = 2034)	
Variable	*n*	%	*n*	%	*n*	%	*n*	%
Gender								
Male	515	46.31	909	45.38	907	45.17	928	45.62
Female	597	53.69	1094	54.62	1101	54.83	1106	54.38
Age								
15–24	170	15.29	299	14.93	280	13.94	334	16.42
25–34	193	17.36	359	17.92	410	20.42	385	18.93
35–44	215	19.33	394	19.67	375	18.68	345	16.96
45–54	220	19.78	387	19.32	323	16.09	344	16.91
55–65	188	16.91	305	15.23	300	14.94	321	15.78
65 or above	126	11.33	259	12.93	320	15.94	305	15.00
Marital status								
Married	634	57.01	1097	54.77	1124	55.98	1117	54.92
Single/divorced/widowed	478	42.99	906	45.23	884	44.02	917	45.08
Education level								
Tertiary or above	567	50.99	1022	51.02	997	49.65	1047	51.47
Secondary	485	43.62	852	42.54	851	42.38	829	40.76
Primary or below	60	5.40	129	6.44	160	7.97	158	7.77
Employment								
Employed	677	60.88	1246	62.21	1212	60.36	1192	58.60
Dependent/unemployed	435	39.12	757	37.79	796	39.64	842	41.40
Monthly household income (HK$)^a^								
$80000 or above	188	16.91	315	15.73	321	15.99	308	15.14
$60000–$79999	111	9.98	188	9.39	168	8.37	183	9.00
$40000–$59999	286	25.72	465	23.22	404	20.12	441	21.68
$20000–$39999	326	29.32	582	29.06	595	29.63	615	30.24
$19999 or below	201	18.08	453	22.62	520	25.90	487	23.94
COVID-19 stress								
Low	1112	100.00	2003	100.00	818	40.74	2034	100.00
High	0	0.00	0	0.00	1190	59.26	0	0.00
Unrest-related distress								
Low	369	33.18	409	20.42	433	21.56	468	23.01
High	743	66.82	1594	79.58	1575	78.44	1566	76.99
Exposure to unrest^b^	98	–	48	–	42	–	97	–
Probable depression^c^								
No	847	76.17	1484	74.09	1722	85.76	1739	85.50
Yes	265	23.83	519	25.91	286	14.24	295	14.50

a US$1 ≈ HK$7.80.

b The number of conflicts/protests (i.e. ‘exposure to unrest’ events) at each timepoints is presented.

c Scores of 10 or above in the 9-item Patient Health Questionnaire (PHQ-9) were used to define probable depression.

### Measures

#### Probable depression

Respondents rated nine items on depressive symptoms *over the past two weeks* on a 4-point scale (0 = not at all, 1 = on several days, 2 = on more than half of the days, 3 = nearly every day) using the Chinese version 9-item Patient Health Questionnaire (PHQ-9) (Yeung *et al*., 2008). Higher scores (range = 0–27) indicated higher levels of depressive symptoms. Scores of 10 or above indicated probable depression (Levis *et al*., 2019). Cronbach's alphas were 0.842, 0.854, 0.850 and 0.827 across the four administrations respectively.

#### Unrest-related distress

Respondents rated to what extent they felt distress over (1) the government's handling of unrest, (2) confrontations between police and protestors, and the use of riot control measures such as physical assault, tear gas, and rubber bullets, and (3) widespread and ongoing demonstrations and protests on a 4-point scale (0 = not at all, 1 = some, 2 = quite a bit, 3 = a lot). Scores of each item were recoded into low (0) and high (1), with low indicating ‘not at all’/‘some’ in all three items and high indicating ‘quite a bit’/‘a lot’ in at least one item. Binary coding of distress relating to large-scale disasters demonstrated statistical sensitivity in detecting important mental health problems in previous studies (Bonanno *et al*., 2007; Hou *et al*., 2022).

#### Exposure to unrest

Exposure to unrest was quantified as the number of conflicts/protests occurred *during the past month* at each timepoint. Event count has been widely used to reflect the objective levels of traumatic stress across previous studies. For example, evidence showed good predictive utility of the number of traumatic events on PTSD among a post-conflict population (Wilker *et al*., 2015) and first responders (Geronazzo-Alman *et al*., 2017). The number of events related to the anti-ELAB movement from June 2019 to July 2020 was extracted from the Armed Conflict Location and Events Dataset (ACLED) (Raleigh *et al*., 2010). ACLED is a database of real-time data on political violence and protest events across the globe. We adopted strict inclusion criteria for data extraction, and counted events as valid only if (1) the item description contained keywords like ‘name of the unrest’ and ‘slogans’ and ‘event name’ related to the anti-ELAB movement and (2) the item involved conflicts/protests as categorized in ACLED. Duplicates were excluded. Event count was the approximate of the potential level of exposure individuals could possibly encounter at that timepoint.

#### COVID-19 stress (Confounder)

COVID-19 stress (T2–T4) was measured with worry for infection or health-related threat, which were further recoded into low (0) and high (1). For details, see Hou *et al*. (2022). All T1 respondents were assigned a score of 0.

#### Demographics

Respondents' age, gender, education level, employment status, marital status, and monthly household income were recorded.

### Analytic plan

The trends of the prevalence of probable depression, unrest-related distress, and number of conflicts/protests across the four timepoints were described. Logistic regression models were used to examine the association of subjective unrest-related distress (‘high’ = 1) with probable depression (PHQ-9 scores:10–27 = 1), controlling for COVID-19 stress and socio-demographics. The association between unrest-related distress and probable depression was then stratified by the number of conflicts/protests across timepoints. Adjusted odds ratio (aOR) with 95% CI indicated the independent association of each correlate with the outcome. All analyses were performed using SPSS (Version 26).

## Results

### Descriptive statistics

The prevalence of probable depression was 23.8% in July 2019, 25.9% in February 2020, 14.2% in April 2020, and 14.5% in July 2020. The prevalence of unrest-related distress was 66.8% in July 2019, 79.6% in February 2020, 78.4% in April 2020, and 77% in July 2020. We extracted a total of 1227 conflicts/protests relating to the anti-ELAB movement between 6th June 2019 and 31st July 2020. There were 98 conflicts/protests in July 2019, 48 in February 2020, 42 in April 2020, and 97 in July 2020.

### Logistic regressions

Controlling for COVID-19 stress and socio-demographics and taking into consideration the potential association between number of conflicts/protests and probable depression, unrest-related distress was associated with 211% increased odds of probable depression (aOR 3.11, 95% CI 2.19–4.43). The positive association between unrest-related distress and probable depression remained consistent and significant across the four timepoints with varying numbers of conflicts/protests (*p* = 0.108–0.686) (Table 2).

**Table 2.** Logistic regression examining the association of unrest-related distress with probable depression, stratified by the number of conflicts and protests across the four timepoints (*N* = 7157)

Variable	Probable depression^a^
	aOR (95% CI)
Gender	
Male	1.0
Female	1.24 (1.09–1.40)**
Age	
15–24	1.0
25–34	1.12 (0.89–1.39)
35–44	0.95 (0.74–1.21)
45–54	0.76 (0.59, 0.98)*
55–65	0.60 (0.47–0.78)***
65 or above	0.71 (0.54–0.92)*
Marital status	
Married	1.0
Unmarried/divorced/widowed	1.38 (1.19–1.60)***
Education level	
Tertiary or above	1.0
Secondary	1.41 (1.23–1.63)***
Primary or below	2.02 (1.52–2.67)***
Employment	
Employed	1.0
Dependent/unemployed	1.08 (0.92–1.27)
Monthly household income (HK$)^b^	
$80000 or above	1.0
$60000–$79999	1.15 (0.87–1.51)
$40000–$59999	1.36 (1.10–1.70)**
$20000–$39999	1.46 (1.18–1.80)***
$19999 or below	1.71 (1.35–2.16)***
COVID-19 stress	
Low	1.0
High	2.48 (1.83–3.37)***
Unrest-related distress	
Low	1.0
High	3.11 (2.19–4.43)***
Exposure to unrest^c^	
July 2019 (98 conflicts/protests)	1.0
February 2020 (48 conflicts/protests)	1.33 (0.88–2.00)
April 2020 (42 conflicts/protests)	0.31 (0.19–0.53)***
July 2020 (97 conflicts/protests)	0.42 (0.25–0.68)***
Unrest-related distress × Exposure to unrest^c^	
Unrest-related distress × July 2019 (98 conflicts/protests)	1.0
Unrest-related distress × February 2020 (48 conflicts/protests)	0.69 (0.44–1.09)
Unrest-related distress × April 2020 (42 conflicts/protests)	0.71 (0.42–1.21)
Unrest-related distress × July 2020 (97 conflicts/protests)	1.12 (0.65–1.91)

*Abbreviations*. aOR, adjusted odds ratio; CI, confidence interval.

*Note*. *p* values are two-sided, * *p* < 0.05, ** *p* < 0.01, *** *p* < 0.001.

a Scores of 10 or above in the 9-item Patient Health Questionnaire (PHQ-9) were used to define probable depression.

b US$1 ≈ HK$7.80.

c The number of conflicts/protests (i.e. ‘exposure to unrest’ events) at each timepoint is presented in bracket.

## Discussion

This study aims to investigate the association of subjective unrest-related distress with probable depression amid and after widespread civil unrest in Hong Kong between July 2019 and July 2020. We also examined whether the positive association between unrest-related distress and depression changed as a function of the number of conflicts/protests over time. Unrest-related distress was consistently associated with higher odds of probable depression, and the association remained significant at different timepoints. Importantly, this association between unrest-related distress and probable depression held in spite of the slight difference in the patterns of distress (*sustained* prevalence) and probable depression (*decreased* prevalence), as this difference could have suggested an increase in the ‘dosage’ of unrest-related distress needed to sufficiently trigger probable depression overtime.

The role of unrest-related distress in predicting probable depression during and after widespread civil unrest could be explained by the cognitive model of stress-related disorders (e.g. depression, PTSD), for the easily accessible traumatic memories and/or the negative appraisals of such memories could underlie the perpetuating psychiatric symptoms beyond the end of objective incidents (Ehlers and Clark, 2000; Creamer *et al*., 2005; Rubin *et al*., 2008; Duyser *et al*., 2020). Rumination has been identified as a prospective predictor of more severe depressive symptoms in a month's time amidst combined social unrest and COVID-19 (February–March 2020) in Hong Kong (Wong *et al*., 2021b).

Our current results are consistent with not only the conclusion that subjective perceptions of traumatic incidents explain depressive symptoms independent of objective lifetime exposure to these incidents (Boals, 2018) but also the notion that mental health consequences following exposure to potentially traumatic events might be fairly minimal in the absence of negative subjective appraisals (Maschi *et al*., 2011; Danese and Widom, 2020). There is evidence showing that only a fraction of objective traumatic events was subjectively experienced by individuals, suggesting a discrepancy between objective events and subjective experiences amid trauma (Creamer *et al*., 2005; Maschi *et al*., 2011; Boals, 2018). A previous study involving burn survivors found that individuals could experience intrusive memories two years after the accident exposure, and whether they cognitively appraised these memories in a negative way was related to the severity of both depressive and PTSD symptoms, over and beyond the adverse consequences brought by burn-related disabilities (Su, 2018). Our current results are generally in line with this. While the trends of depression and number of conflicts/protests were relatively independent, the positive association between unrest-related distress and probable depression remained significant irrespective of the exact numbers of conflicts/protests across time, providing further supportive evidence that mental health during and after political unrest is dependent upon subjective experiences, regardless of objective criteria defining traumatic incidents (e.g. intensity of protests).

Our study has some limitations. First, repeated cross-sectional data could reflect population-level but not intra-personal changes in unrest-related distress and probable depression as the civil unrest unfolded. Second, the current objective measure of conflicts/protests did not take into account heterogeneity in the nature and intensity across these incidents, or individual differences in the actual exposure to conflicts/protests. Finally, probable depression was based on self-report. Future studies would demonstrate a clearer picture of depression using clinical diagnoses/interviews. Notwithstanding the limitations, this study has built upon our own work and further investigated the subjective versus objective pillars of mental health impact following acute traumatic exposures. Accurate screening and effective interventions are needed to complement the existing already-overloaded mental health care system (Galovski *et al*., 2016; Hou and Hall, 2019; Hou *et al*., 2022). The main implication of the current study is that high unrest-related distress could be one core component of continuous mental health assessment and interventions irrespective of the presence/absence of actual incidents. Its utility could last from the acute phase of the unrest to the seemingly peaceful time after the unrest.

## Supplementary material

For supplementary material accompanying this paper visit https://doi.org/10.1017/gmh.2022.27. click here to view supplementary material
