# Supplementary material for: The relation of unrest-related distress with probable depression during and after widespread civil unrest
Source: Glob Ment Health (Camb). 2022 Jul 20;9:322–7. doi: 10.1017/gmh.2022.27 (PMC9806963; doi:10.1017/gmh.2022.27)
Supplement: Supplementary file 1 [file S2054425122000279sup001.docx]

**SUPPLEMENTARY MATERIAL 1** Linear regression examining the association of unrest-related distress with severity of depressive symptoms, stratified by the number of conflicts and protests across the four timepoints (*N*=7,157).

| **Variables^a^** | ***B* (95% CI)** |
| --- | --- |
| **Gender** | 0.259 (0.039, 0.479)* |
| **Age** | –0.303 (–0.385, –0.222)*** |
| **Marital status** | 0.749 (0.501, 0.998)*** |
| **Education level** | –0.554 (–0.760, –0.34)*** |
| **Employment** | 0.175 (–0.064, 0.415) |
| **Monthly household income (HK$)^b^** | –0.278 (–0.368, –0.188)*** |
| **COVID-19 stress** | 1.558 (1.134, 1.981)*** |
| **Unrest-related distress** | 2.569 (1.987, 3.152)*** |
| **Exposure to unrest^c^** |  |
| February 2020 (48 conflicts/protests) | 0.116 (–0.537, 0.770) |
| April 2020 (42 conflicts/protests) | –2.185 (–2.853, –1.518)*** |
| July 2020 (97 conflicts/protests) | –1.960 (–2.594, –1.326)*** |
| **Unrest-related distress × Exposure to unrest^c^** |  |
| Unrest-related distress × February 2020 (48 conflicts/protests) | –0.478 (–1.247, 0.290) |
| Unrest-related distress × April 2020 (42 conflicts/protests) | –1.226 (–1.994, –0.458)** |
| Unrest-related distress × July 2020 (97 conflicts/protests) | –0.116 (–0.869, 0.636) |

*Note*. *p*-values are two-sided, * *p* < 0.05, *** *p* < 0.001. *R*^2^ = .333, *F*(14, 7142) = 63.512, *p* < .001.

^a^The following independent variables were coded into categories: Gender (Male=0, Female=1), Age (15–24=1, 65 or above=6), Marital status (Married=0, Unmarried=1), Education level (Primary or below=1, Tertiary or above=3), Employment (Employed=0, Unemployed/Dependent=1), Monthly household income ($19,999 or below=1; $80,000 or above=5), COVID-19 stress (Low=0, High=1), Unrest-related distress (No=0, Yes=1).

^b^US$1≈HK$7.80.

^c^The number of conflicts/protests (i.e., “exposure to unrest” events) at different timepoints are presented in brackets.
